# Potential Osteoporosis Recovery by Deep Sea Water through Bone Regeneration in SAMP8 Mice

**DOI:** 10.1155/2013/161976

**Published:** 2013-08-06

**Authors:** Hen-Yu Liu, Ming-Che Liu, Ming-Fu Wang, Wei-Hong Chen, Ching-Yu Tsai, Kuan-Hsien Wu, Che-Tong Lin, Ying-Hua Shieh, Rong Zeng, Win-Ping Deng

**Affiliations:** ^1^Stem Cell Research Center, Taipei Medical University, Taipei, Taiwan; ^2^Graduate Institute of Biomedical Materials and Tissue Engineering, Taipei Medical University, Taipei, Taiwan; ^3^School of Dentistry, College of Oral Medicine, Taipei Medical University, Taipei, Taiwan; ^4^Department of Urology, Taipei Medical University Hospital, Taipei, Taiwan; ^5^Department of Food and Nutrition, Providence University, Taichung, Taiwan; ^6^Graduate Institute of Oral Rehabilitation Sciences, Taipei Medical University, Taipei, Taiwan; ^7^Department of Family Medicine, Taipei Medical University, Wan Fang Hospital, Taipei, Taiwan; ^8^Department of Orthopedic Surgery, The Affiliated Hospital, Guangdong Medical College, Zhanjiang 524001, China; ^9^Translational Research Laboratory, Cancer Center, Taipei Medical University, Taipei, Taiwan; ^10^Cancer Center, Taipei Medical University Hospital, Taipei, Taiwan

## Abstract

The aim of this study is to examine the therapeutic potential of deep sea water (DSW) on osteoporosis. Previously, we have established the ovariectomized senescence-accelerated mice (OVX-SAMP8) and demonstrated strong recovery of osteoporosis by stem cell and platelet-rich plasma (PRP). Deep sea water at hardness (HD) 1000 showed significant increase in proliferation of osteoblastic cell (MC3T3) by MTT assay. For *in vivo* animal study, bone mineral density (BMD) was strongly enhanced followed by the significantly increased trabecular numbers through micro-CT examination after a 4-month deep sea water treatment, and biochemistry analysis showed that serum alkaline phosphatase (ALP) activity was decreased. For stage-specific osteogenesis, bone marrow-derived stromal cells (BMSCs) were harvested and examined. Deep sea water-treated BMSCs showed stronger osteogenic differentiation such as BMP2, RUNX2, OPN, and OCN, and enhanced colony forming abilities, compared to the control group. Interestingly, most untreated OVX-SAMP8 mice died around 10 months; however, approximately 57% of DSW-treated groups lived up to 16.6 months, a life expectancy similar to the previously reported life expectancy for SAMR1 24 months. The results demonstrated the regenerative potentials of deep sea water on osteogenesis, showing that deep sea water could potentially be applied in osteoporosis therapy as a complementary and alternative medicine (CAM).

## 1. Introduction

Osteoporosis, a common disease caused by imbalanced bone remodeling, is a global public health problem. Patients with osteoporosis are often accompanied with pain, disability, and decline in the quality of life, rise in mortality, and depletion of local health care budget [1–4]. The major type of osteoporosis occurred in postmenopausal women with an age range of 50–70 [5]. Bone density index rapidly decreased after the menopause, due to sex hormone imbalance and the lack of estrogen. The imbalance of homeostasis between osteoclasts and osteoblasts also leads to increased bone resorption [6, 7]. In addition, senile osteoporosis with increased age and the reduction of growth hormone (GH) which stimulates renal-synthesized 1 alpha,25-dihydroxyvitamin D3 (1,25(OH)2D3) is also another cause of osteoporosis [8, 9]. Age-related bone loss causes bone trabecular thinning as well as the loss of cortical layer of bone tissue, and the cortex becomes porous which will increase the femoral neck fracture rate [10]. For osteoporosis, supplemental dietary calcium and vitamin D may reduce the risk of fractures in postmenopausal women [11]. Drugs, lifestyle changes, home safety, and hip protection are viable approaches for the prevention of osteoporosis. According to the World Health Organization Criteria (WHO) statistics, the penetration rate of osteoporosis in women over the age of 65 is up to 35%; hence, the advanced treatment for osteoporosis has become emergent.

Deep sea water (DSW) generally refers to sea water from a depth of more than 200 meters (m). It could be characterized by its purity, abundant nutrients, and minerals. Currently, DSW has been applied in the ground of food, agriculture, cosmetic, and medical field due to its high contents of unique minerals including sodium (Na), magnesium (Mg), calcium (Ca), potassium (K), zinc (Zn), and vanadium (V) [12]. DSW has been reported to stimulate both osteoblastogenesis and osteoclastogenesis in bone turnover [13]. NaCl from DSW also improves the biochemical properties of bone. DSW combined with soluble silicon as natural material could promote cell proliferation of osteoblast and enhance the osteogenesis-related gene expression in animal studies [13]. The DSW utilized in this study is drawn from the Pacific Ocean at a depth of 662 m and a distance of 5 kilometers (km) off the coast of Hualien County, Taiwan [14, 15], which contains abundant amounts of trace elements, including high concentrations of four essential minerals: Mg (96200 mg/L), K (10800 mg/L), Na (9010 mg/L), and Ca (39 mg/L).

Our previous study has demonstrated that transplantation of platelet-rich plasma- (PRP-) treated NIH3T3-G cells into OVX-SAMP8 mice significantly reversed osteoporosis. We also showed that PRP could not only increase bone regeneration but also reduce bone marrow adiposity in the osteoporotic mice [16–18]. In this study, we investigated the possibility of the treatment of osteoporosis by DSW. For osteoporotic model, SAMP8 mice received bilateral salpingo-oophorectomy at 4 months old and subsequently fed with DSW for 15 days. The effects of DSW on bone regeneration were then analyzed by bone mineral density, micro-CT, bone structure with HE stain, and the activities of isolated bone marrow stromal cells. Here, we demonstrated that DSW not only induced bone regeneration but also strongly recovered bone loss in OVX-SAMP8 mice. In addition, we have observed that DSW would be effective in prevention of osteoporosis and might be a complementary and alternative medicine (CAM).

## 2. Materials and Methods

### 2.1. Characterization of Deep Sea Water (DSW)

DSW was obtained from the Pacific Ocean at a depth of 662 m [14, 15]. The obtained DSW was subjected to filtration to remove microorganism and virus and then concentrated. The elements contained in concentrated DSW were measured by inductively coupled plasma mass spectroscopy. As shown in Supplemental Table 1, the concentrated DSW contained high amounts of several essential minerals such as magnesium (Mg), potassium (K), sodium (Na), and calcium (Ca) (see Supplementary Material availbale online at http://dx.doi.org/10.1155/2013/161976). The final hardness of concentrated DSW was determined as 400,000 mg/L by using calcium and magnesium concentrations.

### 2.2. Cell Line and Cell Viability

Mouse preosteoblast cell line (MC3T3-E1, ATCC CRL-2593) was provided by Dr. Alexander T.H Wu (Taipei medical University, Taipei, Taiwan) and was cultured in 96 well plates (1 × 10^4^ cells/well) in alpha minimum essential medium (*α*-MEM) supplemented with 10% fetal bovine serum (FBS) or/and different concentration of DSW (ranged from 500 to 2000 HD). MTT reagent was added into each well on day 3 of cell growth in culture, and cell viability was detected by Multiskan PC (Thermo Lab). For the cell counting assay, MC3T3 (1 × 10^4^) were seeded in 6 cm dish containing DSW-derived medium (hardness of 1000 mg/L)and incubated at 37°C in 5% CO_2_ atmosphere. After 72 hrs, MC3T3 were collected, and the cell numbers were counted by trypsin and ethylenediaminetetraacetic acid treatment.

### 2.3. Experimental Animals

All the animal experiment protocol was approved by the Institutional Animal Care and Use Committee of Taipei medical University. SAMP8 mice were ovariectomized (OVX) at 4 months of age to induce osteoporosis and then used in this experiments. Mice were grouped into the following (six mice per group): control group (CTRL, receiving PBS) and DSW group (receiving DSW). Both mice were suggested for daily water uptake which is based on a 22 g mouse consuming 5.2 mL water per day. 

### 2.4. Serum ALP, Mg, and Ca Analyses

All mice were sacrificed and the extracted blood specimens were obtained at 4 months. The plasma concentrations for ALP, Mg, and Ca were then determined by a photometric method according to the manufacturer's instructions (Fuji Dri-Chem Clinical Chemistry Analyzer FDC 3500). 

### 2.5. Bone Mineral Density and Micro-CT Analysis

Dual-energy X-ray absorptiometry (DEXA) analysis was used for the measurement of bone mass in the spine, left/right knee, and left/right femurs. BMD (XR-36; Norland Corp.; host software revision 2.5.3, scanner software revision 2.0.0) was performed 4 months after DSW treatment. All mice were sacrificed and their femurs and tibia bones were collected at 4 months for detecting the trabecular bone and bone volume by micro-CT (Skyscan-1076, Skyscan, Belgium). For trabecular bone analysis and 3D imaging, construction was operated at 50 KV, 200 uA, 0.4° of rotation step, 0.5 mm AI filter, and 9 *μ*m/pixel of scan resolution. Each group contains 6 animals. 

### 2.6. Histological Analysis

For histological determination, bone sample was fixed in 10% formalin and decalcified in 14% EDTA for 3 days. Bone sections (10 *μ*m) were subjected to paraffin-embedding and then stained with hematoxylin and eosin (H&E) staining to detect the trabecular bone in the bone tissue.

### 2.7. Isolation of Bone Marrow Cells

Bone marrow stromal cells (BMSCs) were harvested from femurs and tibias of DSW-treated mice and vehicle-treated mice (CTRL) after 4 months treatment. BMSCs were washed out in the bone marrow and centrifuged at 1000 rpm for 5 min. Cells were all cultured in *α*-MEM supplemented with 10% FBS for one week to remove the nonadherent cells and then washed with PBS. The adherent cells, indicated the BMSCs, were collected for ethylenediaminetetraacetic acid treatment.

### 2.8. Cell Proliferation and Colony-Forming Unit of BMSCs *In Vivo*

BMSCs were cultured in *α*-MEM supplemented with 10% FBS and/or DSW treatment for 3 days, and the cell numbers were counted for evaluating their proliferation. For colony formation assay, BMSCs were cultured in maintenance medium after 14 days and then fixed with 4% formaldehyde. Fixed cells were then stained with 0.5% crystal violet in methanol for 10 min, and formed colonies were then counted.

### 2.9. RT-PCR Analysis

Total RNA from BMSCs was extracted using TRIzol regent (Invitrogen Life Technologies). Gene expression levels were measured by RT-PCR. Primer sequences were indicated as follows: bone morphogenetic protein (BMP2): forward primer 5′-GGTCCTTGCACCAAGATGAAC-3′; reverse primer 5′-CAACCCTCCACAACCATGTC-3′, and temperature 62°C; osteopontin (OPN): forward primer 5′-ATGAGATTGGCAGTGATT-3′, reverse primer 5′-GTTGACCTCAGAAGATGA-3′, and temperature 48.8°C; osteocalcin (OCN): forward primer 5′-CAGCTTGGTGCACACCTAAGC-3′, reverse primer 5′-AGGGTTAAGCTCACACTGCTCC-3′, and temperature 55°C; runt-related transcription factor 2 (RUNX2): forward primer 5′-ACTTTCTCCAGGAAGACTGC-3′; reverse primer 5′-GCTGTTGTTGCTGTTGCTGT-3′, and temperature 55°C; glyceraldehyde 3-phosphate dehydrogenase (GAPDH): was used as an internal control (CTRL) forward primer 5′-GCTCTCCAGAACATCATCCCTGCC-3′; reverse primer 5′-CGTTGTCATACCAGGAAATGAGCTT-3′, and temperature, 55°C. PCR products were separated by electrophoresis on 1% agarose gels (Agarose I; AMRESCO) and visualized with ethidium bromide staining.

### 2.10. Statistical Analysis

All results were represented as mean ± standard deviation (SD). Significant differences between the two groups were determined by Student's *t*-test, *P*  value < 0.05.

## 3. Results

### 3.1. Effect of Deep Sea Water (DSW) Hardness on the Cell Growth of Preosteoblast

**Figure 1:**
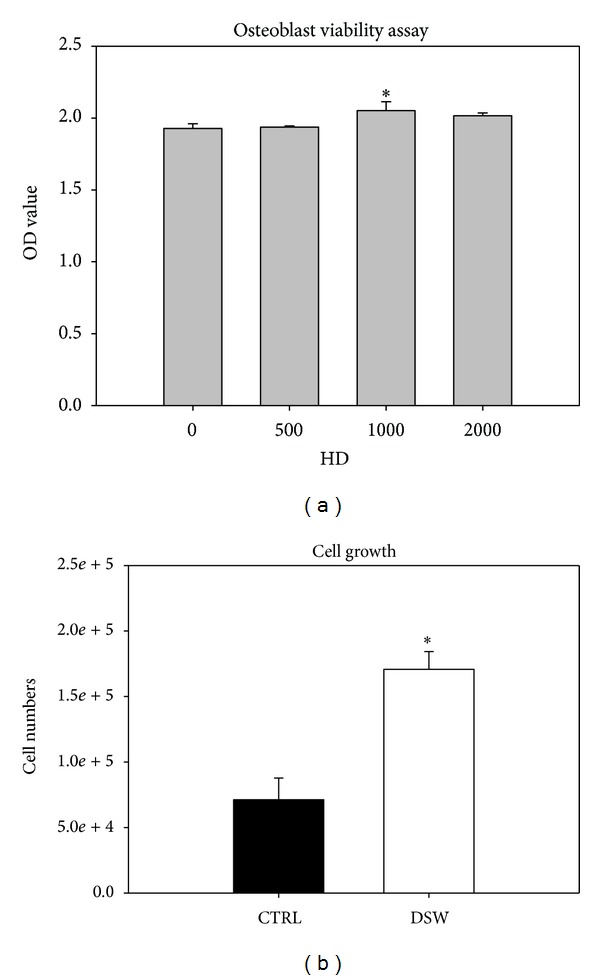
Optimization of hardness of DSW by cell viability of preosteoblast. Comparative proliferation profiles of preosteoblast (MC3T3) in DSW-enriched medium were analyzed by MTT assay (optical densities, mean 6 SD for 3 separate replicates). The MC3T3 cells treated with DSW of hardness (HD) 1000 showed the highest cell viability; *t*-test, *P < 0.05.

To determine the effects of different hardness of DSW water on the cell viability of preosteoblasts, MC3T3 cells were cultured in DSW-derived medium for 48 hrs and analyzed by MTT assay. As shown in Figure 1(a), the DSW with hardness of 50 and 2000 mg/L did not affect the cell viability of preosteoblasts, yet DSW with hardness of 1000 mg/L slightly increased the cell viability of MC3T3 cells. Hence, we further evaluated the cell growth effects of DSW with hardness of 1000 mg/L on MC3T3 cells by using cell-counting assay. After 72 hrs of culture, MC3T3 cells showed 2- to 3-fold enhanced cell growth in DSW-derived medium (hardness of 1000 mg/L) compared to the control medium (Figure 1(b)). These results indicated that 1000 mg/L displays the optimal hardness of DSW for the cell growth of preosteoblasts. Therefore, we utilized DSW with hardness of 1000 mg/L in the following experiments. 

### 3.2. Effect of DSW on Serum Alkaline Phosphatase (ALP), Calcium (Ca), and Magnesium (Mg) Activity in OVX-SAMP8 Mice

**Figure 2:**
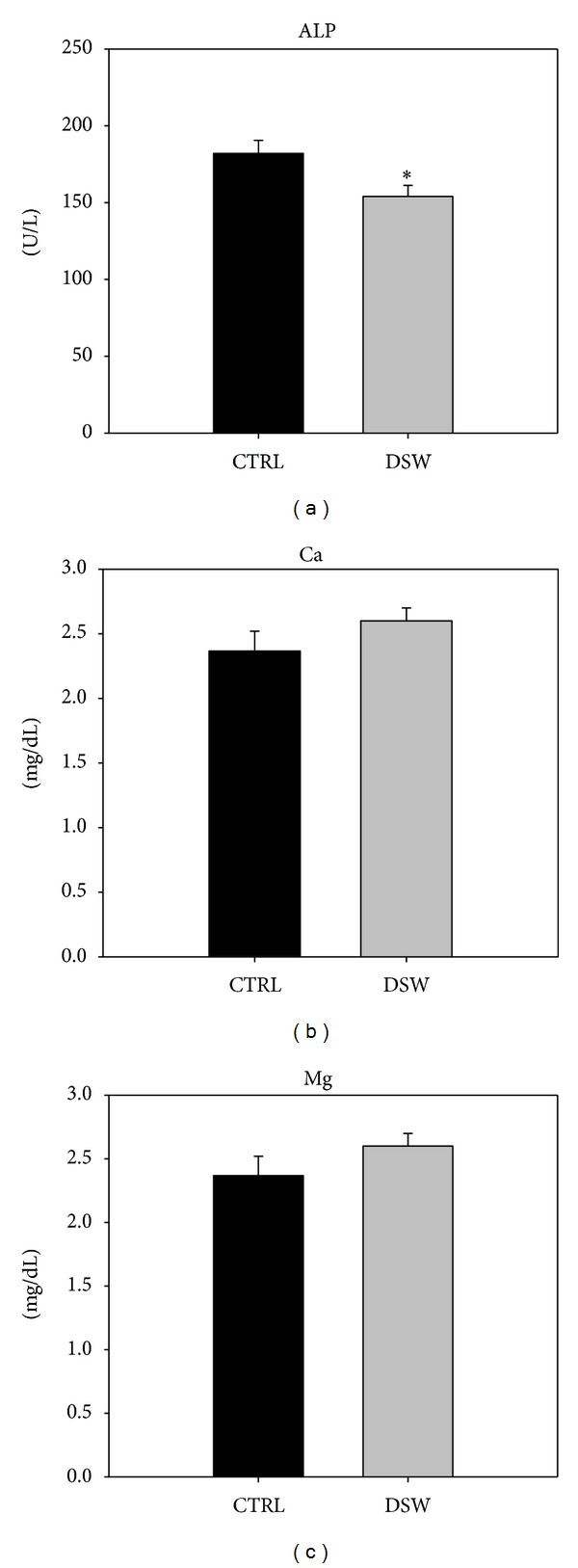
The effect of DSW treatment on serum ALP, Ca, and Mg activity. The serum ALP, Ca and Mg levels after 4 months DSW treatment was measured by ELISA assay. Serum ALP levels presented a significant decrease in DSW-treated mice (a). In contract, Ca and Mg showed no significant different in DSW group compared with control mice ((b) and (c)). Each bar represents the average from six animals; *t*-test, *P < 0.05 versus CTRL group.

Based on the *in vitro* results, DSW of hardness 1000 was applied to OVX-SAMP8 mice for 4 months. We first evaluated the *in vivo* osteogenic effects of DSW by using serum levels of ALP, Ca, and Mg. Serum ALP values reflect the increased turnover associated with bone destruction of aging, menopause, and various conditions affecting bone metabolism. Increased serum ALP levels are associated with an increased risk of rapid bone loss in peri- and postmenopausal women [19–21]. Ca and Mg play important roles in bone homeostasis and metabolism. Decreased serum levels of Ca and Mg have been shown to contribute to the risk of osteoporosis [22–24]. After 4 months DSW treatment, significantly lower ALP activity was evident in the DSW group as compared to control group (Figure 2(a)), which means that DSW could recover the bone loss in OVX-SAMP8 mice. Meanwhile, no significant difference was found in Ca and Mg levels between the DSW and control group (Figures 2(b) and 2(c)). 

### 3.3. Quantitative Analysis of BMD in DSW-Treated Mice

**Figure 3:**
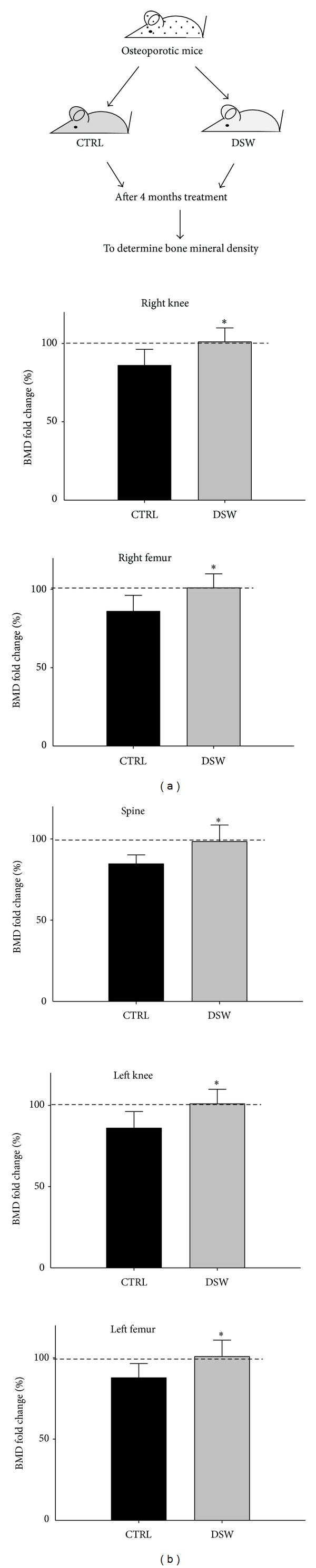
Quantitative analysis of BMD in DSW-treated and control mice. Bone density scores (BMD) of spine, knee (right/left), and femurs (right/left) were measured by Dual-energy X-ray absorptiometry after 4 months treatment (a). DSW-induced bone formation and decreased bone loss were observed in the DSW-treated OVX-SAMP8 mice (b). Values (means + SD) indicate relative BMD levels normalized to month 0 BMD (before operation = 100%; dashed line), respectively; *P < 0.05 determined by *t*-tests; *n* = 6 of each group.

DSW was used for prevention of OVX-SAMP8 osteoporotic mice, and bone density scores of spine, knee (right/left), and femurs (right/left) were determined by dual-energy X-ray absorptiometry (general scheme, Figure 3(a)). As demonstrated in Figure 3, 4 months after treatment, DSW of hardness 1000 significantly improved BMD of OVX-SAMP8 mice, compared with vehicle-treated OVX-SAMP8 mice, indicating that DSW induced bone regeneration and recovered bone mass loss in these osteoporotic mice (Figure 3(b)). 

### 3.4. Photomicrographs for Bone Recovery in OVX-SAMP8 Mice by DSW

**Figure 4:**
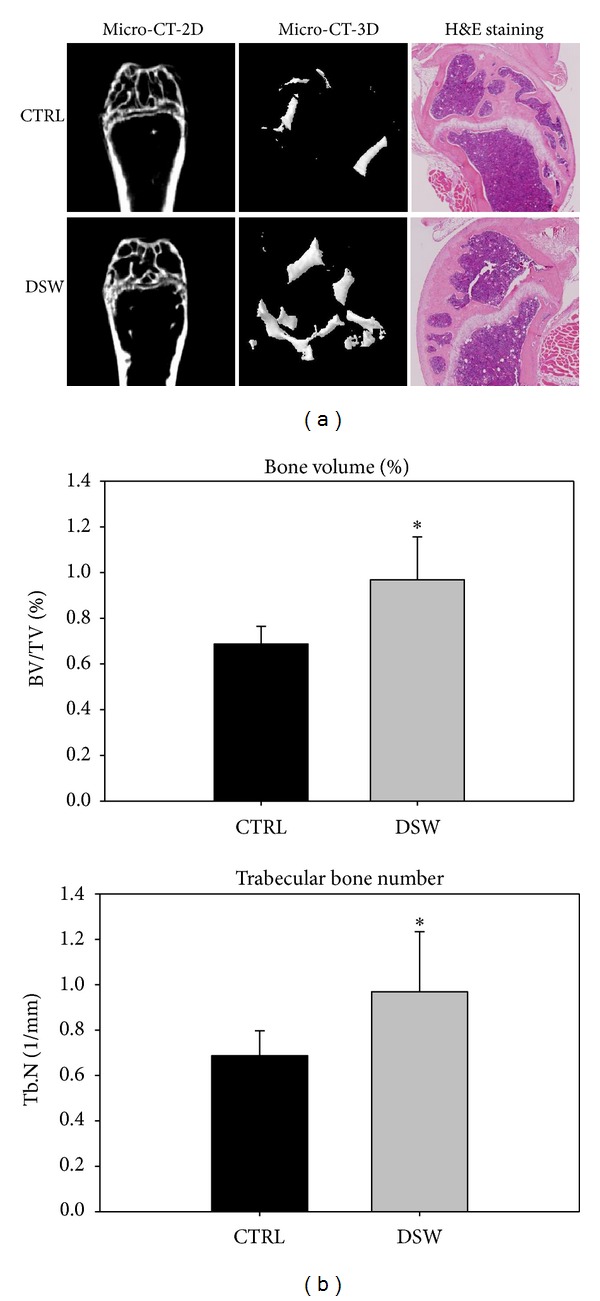
Effects of DSW on bone structure of OVX-SAMP8 mice. Comparative analyses of bone structure between control and DSW-treated OVX-SAMP8 mice. Micro-CT imaging observed that bone structure (trabecular/cortical bone, 2D), trabecular bone volume (3D), and trabecular bone mass were increased in the DSW-treated OVX-SAMP8 mice after 4 months treatment. H&E staining represented a higher ratio of bone mass in DSW-treated mice (a). The volume of trabecular bone (bone volume/total bone volume (BV/TV)), and the numbers of trabecular bone (Tb.N) were calculated (right panel), and bars represented the average from six animals (b); *t*-test, *P < 0.05 versus CTRL group.

The improvement in BMD scores in DSW-treated animals was further supported by bone morphological analysis. Both micro-CT 2D and 3D imaging demonstrated higher trabecular area and volume in DSW-treated OVX-SAMP8 animals (left and middle two columns, resp., Figure 4(a)). Meanwhile, histological sections of bone tissues from DSW-treated mice also showed more trabecular bone areas in the bone marrow (right two columns, Figure 4(a)). After the relative trabecular bone volume ratio to the total bone volume (BV/TV) was measured, it was clear that a higher percent bone volume ratio (upper panel, Figure 4(b)) and trabecular bone number (lower panel, Figure 4(b)) were also found in DSW-treated OVX-SAMP8 mice.

### 3.5. The Proliferation and Colony-Forming Ability of Bone Marrow Stem Cell (BMSC) Derived from DSW-Treated Mice

**Figure 5:**
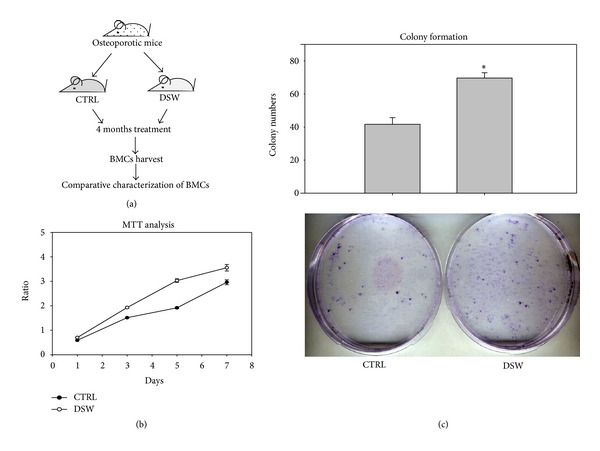
*Ex vivo* proliferation and colony-forming ability of isolated BMSCs. Comparative proliferation profiles of BMSCs from DSW and vehicle-treated mice were demonstrated by MTT assay ((a) and (b)). BMSCs were cultured for 14 days, and their colony forming abilities were assayed. Data was expressed quantitatively in the upper panel (c). Representative results of 3 experiments are demonstrated; *t*-test, *P < 0.05 versus CTRL group.

To validate these observations and determine the effect of DSW on bone microenvironment in OVX-SAMP8 mice, BMSCs in DSW-treated mice were isolated and examined for cell proliferation and colony-forming ability (general scheme, Figure 5(a)). The BMSCs from DSW-treated SAMP8 mice demonstrated significantly higher proliferative activity and higher numbers of colonies as compared to those from vehicle-treated mice after 7 days incubation (Figures 5(b) and 5(c)). This finding suggested that the BMSCs from DSW-treated SAMP8 mice had statistically significant proliferative and colony-forming abilities. 

### 3.6. Effects of DSW on Osteogenic Differentiation of BMSCs *In Vivo*

**Figure 6:**
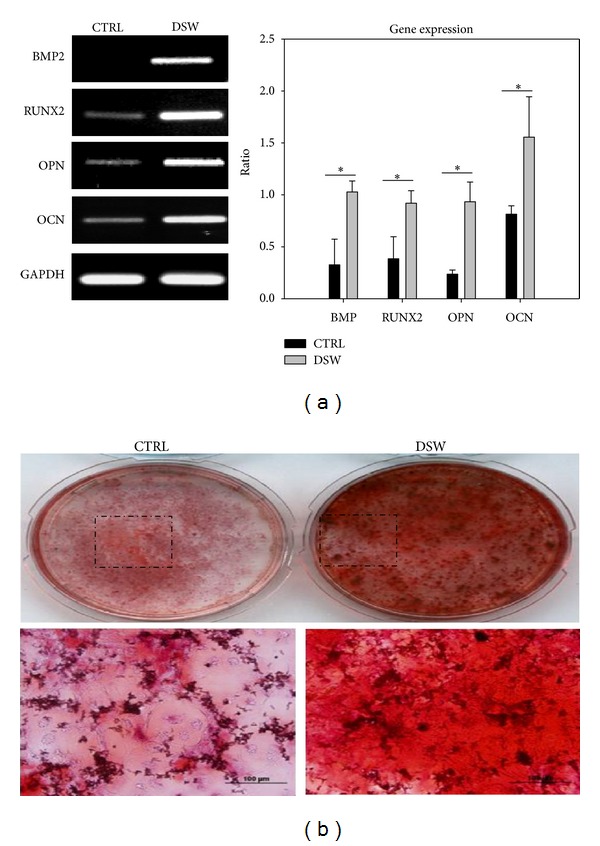
Comparative expression profile of osteogenic differentiation markers of BMSCs in osteoporotic mice. Effect of DSW on osteogenic mRNA expression in specific genes including bone morphogenetic protein 2 (BMP2), runt-related transcription factor 2 (RUNX2), Osteopontin (OPN) and Osteocalcin (OCN) of BMSCs was quantitatively measured ((a), right/left panel). The maturation of osteogenic differentiation was then examined by Alizarin Red S staining (b), and more calcium nodules were formed in DSW group. Representative results of 3 experiments are demonstrated; *P < 0.05 versus CTRL group.

Following the characterization scheme in Figure 5, osteogenic differentiation markers, including BMP2 (regulate the transcription factor Runx2 expression), RUNX2 (an early stage marker for osteogenesis), OPN (an intermediate marker for osteogenesis), and OCN (a late stage marker for osteogenesis) mRNA transcripts of BMSCs from DSW-treated OVX-SAMP8, were examined and compared. The mRNA level of RUNX2, BMP2, OPN, and OCN was significantly upregulated in DSW-treated mice when compare to control mice (Figure 6(a)). Moreover, the Alizarin Red S staining showed an increased level of matrix mineralization in the BMSCs isolated from the DSW-treated group, as compared to the control group (Figure 6(b)). 

### 3.7. Survival of Osteoporotic Mice Treated with DSW

**Figure 7:**
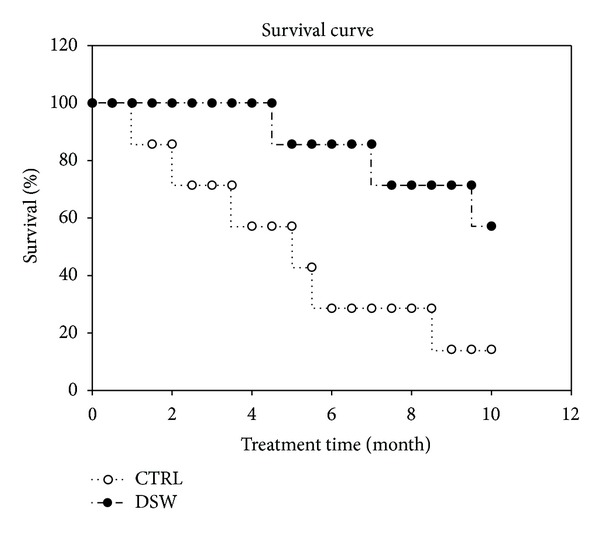
Survival of DSW-treated OVX-SAMP8 mice. The Kaplan-Meyer survival plots of mice from DSW (-●-) and vehicle groups (-○-) were shown.

In addition to significantly improving bone trabecular architecture and BMD, the DSW could extend the life span of OVX-SAMP8 mice, which was markedly longer than that of the untreated OVX-SAMP8 (Figure 7). Most untreated OVX-SAMP8 mice died around 10 months; however, approximately 57% of DSW-treated groups lived up to 16.6 months, a life expectancy similar to the previously reported life expectancy for SAMR1 24 months [16].

## 4. Discussion

Previously, we have demonstrated the osteogenic regeneration mechanism by stem cells and platelet-rich plasma in OVX-SAMP8 mice [16]. In this study, we extended to examine the feasibility of using deep sea water (DSW) as a molecular cocktail to modulate the osteogenesis in osteoporotic mouse model. DSW contained major trace elements such as magnesium (Mg) and calcium (Ca), that have been reported to regulate bone metabolism. Mg and Ca were also found to induce murine osteoblast differentiation in MC3T3 cells. When MC3T3 cells were cultured in Mg- and Ca-free medium, their osteoblast differentiation marker osteocalcin (OCN) was reduced [25]. Epidemiologic studies showed a good correlation between Mg/Ca and bone density, in which low Mg and Ca intake might decrease bone mass [26, 27], bone turnover, and also cause high risk of fracture and osteoporosis. Moreover, the hardness (HD) of DSW which resulted from the composition of Mg and Ca also reported to modulate osteogenesis [28, 29]. In this study, our result showed that DSW of hardness 1000 (1000HD) appeared to be the optimal condition for osteoblast proliferation (Figure 1) and was applied for later experiments.

Serum alkaline phosphatase (ALP) is a group of enzymes found primarily in bone tissue. The primary importance of measuring ALP is to check the possibility of bone disease such as fractures and bone loss [30, 31]. The decreased serum ALP activity and bone mass recovery were found in early postmenopausal woman with osteoporosis after hormone replacement therapy [32]. From the biochemical analysis, OVX-SAMP8 mice exhibited decreased ALP activity after 4 months DSW treatment (Figure 2). The decreased ALP activities were evidenced by bone mineral density (BMD) which indicated that bone quality in OVX-SAMP8 mice was enhanced by DSW (Figure 3). Interestingly, there was no significant difference in Ca and Mg levels after DSW treatment compared to control group. In the present study, OVX-SAMP8 mice exhibited increased BMD and decreased bone loss after 4 months treatment with DSW. The histological and micro-CT 2D and 3D imaging data also demonstrated that BV/TV ratio, trabecular bone numbers, and volume were strongly enhanced in DSW-treated OVX-SAMP8 animals (Figure 4). BMD has been shown to be an important factor to determine the strength of cancellous bone and trabecular bone [33, 34]. In addition to BMD, trabecular structural parameters, such as fractal dimension or tissue volume, could enhance the prediction of bone mechanical quality [35, 36]. These analyses indicated that DSW treatment reduced the bone loss and induced the bone recovery.

Interestingly, we initially used *in vitro* osteoblast cell culture to determine the effects of DSW on cell proliferation and colony formation; however, no effects were found (data not shown). Metabolism would be an important factor to be considered. Therefore, an alternative approach was designed and shown in general scheme of Figure 5(a). After administration of DSW, BMSCs in DSW-treated mice were then isolated and examined for cell proliferation and colony-forming ability. BMSCs obtained from DSW-treated mice showed markedly induction in osteogenesis-related molecules including BMP2, RUNX2, OPN, and OCN (Figure 6). BMP2 has been shown to regulate osterix via RUNX2 [37], an essential transcription factor for osteoblast differentiation [38]. During osteogenesis, OPN is an intermediate marker, while OCN is a late stage marker of osteoblasts [39]. DSW was found to strongly modulate the molecular determinants in bone microenvironment for osteogenesis. Meanwhile, the higher cell growth and colony formation were also detected in bone marrow cells isolated from DSW-treated mice (Figure 5). Taken together, DSW treatment enhanced bone formation in OVX-SAMP8 mice through metabolism.

 Our data demonstrated that therapy with DSW appeared to prolong the life span of OVX-SAMP8 mice (Figure 7). Previously, we have shown that cell therapy with growth factors (PRP/NIH3T3-G) prolonged the life span of the osteoporotic mice through the bone mass recovery [16]. Epidemiologic studies indicated that the high mortality of hip fractures patients was 3-fold higher than those of general population. In contrast, the increased bone mass can also reduce the risk of bone fracture and subsequently increase the life expectancy in osteoporotic patients [40–42]. DSW treatment strongly indicated that not only bone mass was recovered but also osteogenesis was induced in osteoporotic mice and then potentially improving (regenerating) their structure and function in degenerative lesions, aging tissue, and organs. Furthermore, DSW treatment could be applied for rejuvenation therapy and bone regeneration for prolongation of life.

## 5. Conclusion

In this study, the potential effects of DSW on bone regeneration in osteoporosis recovery were investigated; we found that DSW promoted osteoblast viability and increased BMD scores, trabecular bone numbers, and ameliorated symptoms of osteoporosis. These findings suggest that DSW could be a useful treatment for preventing osteoporosis in the near future.

## Supplementary Material

DSW was analyzed in SGS Taiwan Environmental Lab (Taipei, Taiwan) and used the inductively coupled plasma mass spectroscopy (ICP-MS) to determine the trace elements in Deep sea water (DSW). The following Supplemental Table 1 shows the concentrated DSW contained abundant amounts of elements such as magnesium (Mg), potassium (K), sodium (Na), and calcium (Ca).

## Abbreviations

DSW: Deep sea water
BMD: Bone mineral density
BMSCs: Bone marrow stromal cells.
