# Supplementary material for: Potential Osteoporosis Recovery by Deep Sea Water through Bone Regeneration in SAMP8 Mice
Source: Evid Based Complement Alternat Med. 2013 Aug 6;2013:161976. doi: 10.1155/2013/161976 (PMC3773439; doi:10.1155/2013/161976)
Supplement: Supplementary file 1 — DSW was analyzed in SGS Taiwan Environmental Lab (Taipei, Taiwan) and used the inductively coupled plasma mass spectroscopy (ICP-MS) to determine the trace elements in Deep sea water (DSW). The following Supplemental Table 1 shows the concentrated DSW contained abundant amounts of elements such as magnesium (Mg), potassium (K), sodium (Na), and calcium (Ca). [file 161976.f1.pdf]

Table 1. Elements content in DSW

Elments	DSW (mg/L)
Magnesium	96200
Potassium	10800
Sodium	9010
Calcium	39
Fluorine	22.4
Lithium	17.1
Silicon	2.48
Strontium	0.55
Molybdenum	0.47
Nickel	0.05
Zinc	0.0038
Iron	0.0033
Manganese	0.0016
